# Managing hospital doctors and their practice: what can we learn about human resource management from non-healthcare organisations?

**DOI:** 10.1186/s12913-014-0566-5

**Published:** 2014-11-21

**Authors:** Timothy M Trebble, Nicola Heyworth, Nicholas Clarke, Timothy Powell, Peter M Hockey

**Affiliations:** Department of Gastroenterology, Portsmouth Hospitals NHS Trust, Queen Alexandra Hospital, Portsmouth, PO6 3LY UK; Department of Human Resources, Heatherwood and Wexham Park Hospitals NHS Foundation Trust, Slough, SL2 4HL UK; Department of Human Resources, School of Management, University of Southampton, Southampton, SO17 1BJ UK; Department of Human Resources, Portsmouth Hospitals NHS Trust, Queen Alexandra Hospital, Portsmouth, PO6 3LY UK; Health Education Wessex, Otterbourne, Winchester, Hampshire SO21 2RU UK

**Keywords:** Performance management, Clinicians, Human resource management, Performance improvement, Performance improvement, Organisational studies, Service evaluation

## Abstract

**Background:**

Improved management of clinicians’ time and practice is advocated to address increasing demands on healthcare provision in the UK National Health Service (NHS). Human resource management (HRM) is associated with improvements in organisational performance and outcomes within and outside of healthcare, but with limited use in managing individual clinicians. This may reflect the absence of effective and transferrable models.

**Methods:**

The current systems of managing the performance of individual clinicians in a secondary healthcare organisation were reviewed through the study of practice in 10 successful partnership organisations, including knowledge worker predominant, within commercial, public and voluntary sector operating environments. Reciprocal visits to the secondary healthcare environment were undertaken.

**Results:**

Six themes in performance related HRM were identified across the external organisations representing best practice and considered transferrable to managing clinicians in secondary care organisations. These included: performance measurement through defined outcomes at the team level with decision making through local data interpretation; performance improvement through empowered formal leadership with organisational support; individual performance review (IPR); and reward, recognition and talent management. The role of the executive was considered essential to support and implement effective HRM, with management of staff performance, behaviour and development integrated into organisational strategy, including through the use of universally applied values and effective communication. These approaches reflected many of the key aspects of high performance work systems and strategic HRM.

**Conclusions:**

There is the potential to develop systems of HRM of individual clinicians in secondary healthcare to improve practice. This should include both performance measurement and performance improvement but also engagement at an organisational level. This suggests that effective HRM and performance management of individual clinicians may be possible but requires an alternative approach for the NHS.

## Background

Secondary healthcare accounts for a majority of National Health Service (NHS) spending [1] within which speciality based clinicians represent a significant human capital resource, cost and determinant for quality and productivity. However, increased demand for specialist healthcare in the context of limited financial resources, [2,3] and supported by evidence of variation in their productivity and practice [4-6] has led to a focus on effectively managing clinicians to optimise their performance in line with organisational needs [3].

Human resource management (HRM) relates to systems of improving utilisation of human capital and associated productivity through developing relationships and objectives of employment between staff and their organisation [7,8]. HRM is considered to be strategic when it both directly supports achieving organisational objectives and is underpinned by a theoretical framework [9]. Examples of such frameworks include: “universalist” or best practice as a single optimum system of managing staff irrespective of operating environment; [10] “contingency” that reflects the needs of the local operating environment integrated vertically and externally e.g. business strategy, and horizontally and internally e.g. staff working practices (either behaviour or systems based); [11] resource-based that considers staff and their skills as a resource to be developed for competitive advantage, rather than simply supporting an organisation in its aims; [12] finally, transactional, that aims to manage staff based through extrinsic motivational approaches.

HRM can be considered relevant to managing the performance of speciality based clinicians in secondary healthcare from a resource-based perspective consistent with their potential status as valuable, limited in availability, imitable and non-substitutable [12]. Furthermore, HRM practices are associated with positive effects on the behaviour of healthcare staff where they enhance autonomy and employee participation in change [13]. Performance management, a system of HRM for managing individual staff through feedback, evaluation and participatory goal setting, is associated with a positive effect on staff behaviour and attitudes and improvement in indicators of clinical care [13-15]. Finally, introducing “bundles” of progressive HRM practices within a framework for managing healthcare staff, known as high performance work systems, are associated with improved delivery of care [16].

HRM practices have been advocated for clinicians [3,17] in job planning, [18] reward and remuneration, [19] and quality assurance, [20] but have been associated with poor clinical engagement, [17,21,22] and criticism of their processes and indicators, [23] leadership, [24,25] data validity [26] and quality of local information systems [27] involved. Secondary care clinicians in the NHS continue to be managed through a nationally implemented contract based on time contribution alone [28] and associated with a reduction in productivity since implementation [29] whereas productivity improvement has traditionally used financial incentivisation with uncertain and possibly detrimental long term consequences [30,31] including demotivation of staff, [31] dysfunctional behaviour, [32] poorly sustained improvements in productivity and practice [19] and conflicting opinions on benefits and consequences for patient care [33-35]. Moreover, performance related HRM strategies incorrectly applied, including in healthcare, may lead to unintended consequences such as gaming, [33,34] “resistance to change” [22] and erroneous conclusions [36].

There is a paucity of published examples of successful models for managing individual clinicians in secondary care with only limited sustained benefit demonstrated from clinical audit, feedback and education [36,37] and conflicting results of productivity based interventions in clinical practice [38,39]. There is therefore a need for new and effective systems of HRM that take into account these factors. The transferability of performance improvement strategies from external organisations into healthcare organisations has been proposed based on advocated similarities in management practices and response to change [25,40,41] and that successful HRM is reflective of good practice independent of its operating environment in the commercial or public sector [32,42]. However, it is also argued that the public sector represents differences in complexity, leadership and influence of external bodies [41] and that healthcare employs a high proportion of medical specialists who, as “knowledge workers”, have specific needs in terms of managing motivation, organisational commitment and other aspects of HRM [43]. These are consistent with the best practice and contingency theoretical frameworks of strategic HRM respectively.

Therefore, the aims of this study were to evaluate current systems of performance related HRM, including strategic HRM and performance management, in successful commercial, public and voluntary sector organisations, including knowledge worker predominant and in diverse operating environments to identify common themes in practice and their transferability to the management of clinicians in a secondary healthcare organisation.

## Methods

### Study design

This was a service evaluation and improvement project reviewing current systems of HRM of individual clinicians in an NHS secondary healthcare organisation through a qualitative study of practice in external partnership organisations. Organisations were identified across the commercial, public and charity sectors, based where possible local to the Trust (in order to reflect similar staff demographic factors). Five of the organisations employed high numbers of specialist, knowledge based employees, consistent with the position of clinicians in secondary care.

Organisations were formally approached with a brief overview of the intended aims of the project. The visiting healthcare team included a physician (and divisional level medical manager) and a clinically trained senior medical human resource manager. Other attendees included human resource management trainees and junior doctors. Commercial sector meetings were hosted by senior managers working at supra-regional level (3 organisations), senior local HR managers (2 organisations), and a senior production plant manager. Public sector meetings involved senior local organisational HR managers with senior general managers up to executive level. The charity sector meeting was undertaken with the chief executive and executive head of HR.

Visits lasted half a day and involved face-to-face, semi-structured interviews relating to HRM and performance management systems. The study team additionally attended shop floor-management meetings and recruitment and interview events, that allowed discussion with non-management employees. Relevant documentation relating to HRM processes were reviewed where available. The study organisations and healthcare teams met between 1 and 5 times, with written notes from the team members amalgamated and triangulated with published sources of information.

All organisations were offered reciprocal visits to the secondary healthcare organisation, that were undertaken by senior HR and regional or supra regional managers from 3 commercial and 2 public sector organisations, and involved half day meetings with presentation of findings of the organisational visits, identification of best practice and discussion of transferability into secondary healthcare. These were hosted by the study team with senior hospital HR and medical managers to and including executive level (Medical director and director of Human Resources). Where not possible post-visit reports were submitted to host organisations for clarification of findings, and resubmitted subsequently following description of the model and study conclusions. Full discussion of the findings of the study were undertaken following presentations to the full executive and senior medical boards at the hospital Trust.

The study was undertaken as a service evaluation meeting criteria for operational improvement activities exempt from ethics review.

#### Study organisations

The study was undertaken at and by employees of Portsmouth Hospitals NHS Trust (PHT) a large secondary healthcare provider to a population of 650,000 in Hampshire, UK, employing approximately 6000 staff of whom 373 were senior clinicians. The organisational visits took place between November 2011 and March 2013. Ten external organisations were included in the study:

1. Six commercial organisations with a reputation for successful business practice, advanced human resource management and effectively adapting to changing external environments and financial challenges [25,44-47].
  - Three organisations, ASDA, John Lewis Partnership (JLP)-John Lewis Stores and JLP-Waitrose, operate nationally within the retail sector with diverse and high turnover product ranges, an advocated focus on quality and direct customer contact, but differing in their organisational structure and commercial focus. JLP employs 85,000 “partners” with total annual sales of over £10 billion, is the UK’s largest example of worker co-ownership [48] and an example of excellence in organisational management [25]. ASDA is one of the largest food (and non-food) retailers in the UK with over 500 stores, total annual sales over £19 billion, employing 180,000 “colleagues”, a subsidiary of Wal-Mart (one of the world’s largest retailers) [49], and uniquely amongst comparable organisations, has been repeatedly identified as one of the best large companies to work for in the UK (Sunday Times Best Big Companies to work for) [50].
  - Three commercial organisations, IBM, Siemens and The MINI Plant, Oxford (MINI), were identified from heavy manufacturing, electronic engineering, technology and IT. All were part of global corporations that were established prior to the inception of the NHS and listed amongst the world’s most high value brands, with financial turnovers approximating to that of the NHS and employing between 100,000 and 450,000 employees [51]. Their employee base, particularly in IBM and Siemens, included high proportions of knowledge workers.
2. Three UK public sector organisations that varied in operating environments, but were all knowledge work and worker based. These included:
  - Ordnance survey [52] (OS) is the national mapping authority of the UK. Formerly a government owned civilian organisation, it reorganised as an autonomous “Trading fund” in 2000. The organisation employs 1200 staff, mainly in a single centre and with a profit making turnover of £120 million per year. OS has a reputation for both high innovation and award winning personnel management [53], with a knowledge worker base in surveying, data capture and management, and product research and development.
  - Office for National Statistics, (ONS) [54] is a public sector body lead by a senior Civil Servant, reporting to Parliament, independently of the Government. It is the UK's largest independent provider of official statistics and the recognised statistical institute for the UK with a leading role in national and international good practice. ONS employs approximately 3000 employees with an annual turnover of £200 million. It has an HR focus on training and developing staff, work life balance and well-being with very high level of staff retention (resulting in an annual staff turnover of 1% compared to a national average of 12.5%). Their knowledge worker base includes statistical data analysts and managers.
  - National air traffic services (NATS) provides air traffic control services within the UK and now across 31 countries, contracting through open national and international competition. It transferred from full public ownership to a public private partnership, independent of the UK Government, in 2001. NATS is a high innovation organisation and world leader in quality and safety, being the first air navigation service provider internationally to develop and adopt a formal safety management system. Today, it handles over 2 million flights per day with attributable delay in 0.1% (compared to a European average delay of 4%). NATS knowledge worker base includes over 900 air traffic controllers amongst a total of 4,500 employees worldwide, with an annual turnover of £900 million (becoming self-financing in 1977 [55]). It has an award winning focus on employee management, including being listed previously as a top organisation to work for in the UK (Sunday Times Best Companies to Work for) [56].
3. A voluntary sector organisation, The Prince’s Trust [57] is a high visibility national charity providing practical and financial support to disadvantaged young people aged 13–30. This has totalled 750,000 people since 1976, and 46,000 in the current year with a 76% positive outcome in terms of helping young people to obtain work, education, self-employment or training. It now has an international advisory capacity. The Trust has an annual turnover of approximately £60 million, predominantly as voluntary income, with 1,000 paid employees and 6000 volunteers (that have considerable similarities to knowledge workers in terms of management) [43]. In 2004, the organisation started a process of adaptation to the changing economic climate and the need for rationalisation and organisational development that included the use of some commercial models of management. In 2011 it merged with another charity, Fairbridge, requiring further organisational development.

## Results

**Figure 1:**
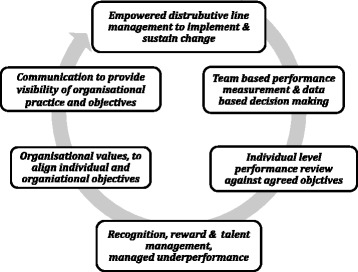
**HRM themes from organisational studies considered transferrable to healthcare organisations.**

Six themes were identified across all organisations that were considered relevant to managing speciality based clinicians in secondary care and directly related to HRM practices such as performance management and strategic HRM (Figure 1). Theoretical saturation was realised prior to completion of the study.

### Performance evaluation and performance based decision making

Performance evaluation can be described as the practice of assessment of individuals, teams or organisations in terms of tangibles such as productivity and quality of work against agreed objectives or expectations and intangibles including practice, behaviour, attitudes and commitment. For employees this may involved comparison of their performance against the contractual duties and responsibilities of their role and in association with competency assessment (knowledge and skills) components of appraisal or individual performance review (IPR) [58]. In the study, performance evaluation was a universal theme across all organisations, with three sub-themes. First, individual level numerical (tangible) performance data, relating to individual activity, was very limited across any of the organisations, and restricted to only a number of specific roles, e.g. sales in the corporate organisations. By comparison, measuring operational performance at a team or section (e.g. less than 12 members of staff), department or unit level was seen as essential practice for transparency and visibility, and was most notable within organisations accustomed to high flow processes, e.g. food retailers, ASDA and John Lewis Partnership Waitrose Food Stores (JLP-Waitrose) and MINI.

Secondly, data was collected for a defined purpose and for decision making, not on the basis of availability e.g. data was for “steering not reporting” (MINI). Comparative data was released to managers reflecting their role and needs e.g. cross site performance indicators for regional managers and shop floor team data for section leaders.

Thirdly, there was both accountability and ownership of performance data by managers down to section or team level even where provided externally. Data could be challenged if felt to be erroneous but there remained confidence in its validity by staff.

The collection of data was automated through electronic databases providing electronic or printed reports, but paper based tables and manual data entry and collation were not seen. At both JLP-Waitrose and ASDA performance data was provided to store managers corporately and included a small number of key indicators (e.g. sales, waste), that could be broken down to departmental level, tracked over time, and in the form of league tables and other forms of cross site comparisons. Within the production line at MINI real time data collection and tracking was used, but a more common system exemplified by the food retailers JLP-Waitrose and ASDA was performance tracking over weeks to months with retrospective and cross site comparisons for benchmarking. The use of heat maps or traffic light annotation to identify high and low performance was common.

The potential risks of over-dependence on performance data for decision making, particularly at the individual level, was recognised by both managers and shop floor staff with the risk of inducing “fear” amongst employees and dysfunctional behaviour (JLP-Waitrose). An advocated reason related to the concerns of evaluating an individual’s performance based on data that was inaccurately or incorrectly interpreted due to unidentified confounding factors and with “tight coupling” [59] of data to management decisions. A further concern was the risk of “data overload”, for example, the MINI production facility had the capability to measure performance data exhaustively but restricted key indicators to only 11 tracked relevant core measures, considered essential by the production manager to provide visibility of activity and potential problems.

Performance data was reviewed frequently and openly with staff both internally e.g. during weekly store management (ASDA) or monthly production team (MINI) meetings, and during routine visits by regional or national managers. But these were considered opportunities for discussion, problem solving and planning with no apparent use of “tight coupling” [59] to management decision, allowing flexibility in response. There was no evidence in any of the organisations of target setting with punitive consequences or direct comparison of heterogeneous data using formula-based composite scores. This contrasted with practice in healthcare [60].

### Empowered supported formal line management

The value placed on effective leadership and management by the organisations, including the need for investment in training and dedicated time, was a key theme. The management approach commonly adopted, and particularly in the retail organisations, was one of support and development. Shop floor staff considered this essential for their merit based management promotion systems to work, and that resulted in younger staff managing those with many years of employment (JLP-Waitrose). Line management was used to connect strategy and implemented practice and involved managers understanding the roles of direct reports, supported by senior support (with bypassing of line managers by staff considered poor practice) (ONS and NATS).

Across the organisations, there was an emphasis on staff “taking personal responsibility for the organisation’s performance” (MINI) and managers leading by example. At MINI it was advised that a manager’s role is to sustain and embed change and that “managers have to believe (in) it”. In ASDA managers were visibly engaged with shop floor company policies e.g. switching off of lights on leaving meeting rooms (“Save £1 and Sell £1”). This was supported by strategies to engage staff and proactively obtain their feedback through the use of electronic staff surveys and workplace based meetings and providing senior managers with a voice (see below).

Recruitment to management roles was often through self-selection followed by structured assessment (public sector organisations and ASDA). Compulsory and structured management training was universal, but particularly visible across the public sector organisations that had introduced part time, on-line and classroom-based leadership programs for all new managers and lasting several months, with coaching used to aid development if required (NATS). This aimed to provide managers with the right skills and to develop independent and capable managers without a dependency on the HR department (OS). ASDA and JLP-John Lewis used dedicated local training stores or centres where staff and permanent dedicated staff were seconded or based. Managers were routinely evaluated using regular, anonymous feedback by direct reports incorporated into their individual performance review (JLP-John Lewis), through facilitated, formative HR supported face to face meetings (Siemens), using capability models (Siemens Leadership Framework), or using multimodal formal evaluation (including psychometric testing) (OS).

An engaged, strategic and decisive executive team was considered essential by all organisations for changing culture and driving performance. The introduction of performance management in the Prince’s Trust was considered to have been dependent on culture change within the executive itself, “we didn’t do anything different but spoke differently”. In two cases, board level decisions had been taken to introduce first name terms across their organisations to encourage more open discussion. Senior managers were visible to staff, walking shop floors to look at produce displays and talking to local managers in retail organisations, visiting assembly lines at MINI to talk to teams on site, to the distribution of individual copies of organisational strategy by hand from regional directors to staff in the Prince’s Trust. In IBM the use of electronic systems of communication was used to keep staff engaged with the local and corporate management.

### Individual performance review with actions

Throughout the studied organisations, appraisal was defined as individual performance review (IPR) with objective setting, talent management and behaviour assessment. Although organisations differed in the comprehensiveness of this process, ranging from all staff to the management stream alone, it was accorded a central role in addressing performance and given appropriate resourcing in terms of time and training (both initial and on-going as refresher courses).

These systems included both the tangibles, e.g. the “what” or outcomes, and the intangibles, the “how” or processes of delivery. Although both were used in commercial sector organizations, “soft”, competency based systems reviewing behaviour, team working and leadership were predominant, and including subjective information, e.g. staff behaviour during mystery shopper’s visits and customer feedback. By comparison, the use of tangible data (for example sales, waste, absenteeism) was evident predominantly in the evaluation of managers. Similar, objective based systems were noted in the public sector, e.g. ONS and voluntary sectors, e.g. the Prince’s Trust.

Objective setting in IPR was considered essential throughout all organisations, as “without objectives there is no clarity in staff roles” (ONS). Other common findings in IPR systems included the aims of aligning individual and organisational objectives, behaviour and values (particularly in IBM, Prince’s Trust and the JLP-John Lewis), and the formal use of an objective based underperformance process where inadequate individual performance was noted. This commonly involved an intensive human resources supported performance process, supervised by the line manager, with regular (e.g. monthly) objective based reviews, retraining and evaluation of skill mix. This was commonly associated with missed pay increments and annual bonuses, potentially leading to disciplinary boards and dismissal if the employee was considered to have failed to address the agreed objectives. However, in all but two organisations dismissal was considered as an exceptional event with re-engagement of staff and improved skills the principle aim and where this was not achieved staff typically left by mutual agreement for alternative employment. More challenging approaches, e.g. “forced distribution” (poorest performing staff automatically starting a performance pathway) were only used in one organisation.

In addition to the annual or biannual formal IPR, the appraisal process was seen as representing a continuous system of awareness of an employee’s performance, with regular informal one to one discussions and tracking of performance data, identifying and addressing performance issues as they occurred. It was advocated that “there should be no surprises when staff and line managers formally meet” (ONS).

IPR was considered the role of line managers but supported by their organisations. In Siemens this included annual “round table discussions” by groups of line managers of their direct reports’ evaluation, including the latter’s wider attitudes to their performance, in order to “calibrate” decisions, provide consensus oversight, guidance to line managers and validation of the IPR result and the system used. In addition an appeals system was available across all organisations but disagreements were commonly handled by line manager directly, and in such instances without interrupting the actions arising following the identification of poor performance. By comparison, 360 degree evaluations were occasionally used to aid personal development but not IPR other than where staff were geographically remote from their line manager (IBM).

### Valuing, developing and retaining staff using reward and talent management

A final common theme related to promoting staff well-being, recognising and valuing their contribution to their organisation and ensuring “staff enjoyed what they do” (NATS). This was noted across all sectors and considered to lead to staff satisfaction, a strong work ethic, organisational commitment, and in attracting and retaining high quality and committed employees when faced with a competing organisation in some cases offering higher wages.

For example, at JLP, where shop floor staff openly advocated “if you cut us we bleed green” (e.g. resembling the colour of the brand label), there was investment in staff leisure and holiday facilities, allowing sabbatical breaks and support for personal development of education, skills and interests outside the working environment. Similarly, IBM invested time and staff resources to support on site social clubs within the organisation promoting diversity of staff to encompass all ethnicities, genders, sexual orientations and social and family circumstances; there was also an emphasis on flexible working and working from home (the “virtual” office) with “fluid” and “flexible” working patterns, that made posts more attractive to staff and fitted with their preferred lifestyles. The Prince’s Trust had instituted the ‘3 Ps’ of Purpose (40%), People (40%) and Performance (20%) with the view that “if the first two are valued and right, then the third should naturally improve”. OS organised “hack days” allowed specialist technical staff to work as they wished with the organisation’s data, although this had additional benefit of developing innovation.

Pay and reward structures had considerable similarities across the organisations, commonly involved a unified banded salary structure, with incremental increases for individual staff addressed at performance review, and automatic increases in only one (public sector) organisation. Incremental pay scales within bandings were performance related and accounted for a difference of up to 60% or more across a band. Staff could voluntarily request downgrading in their pay band and role without prejudice.

Individual performance related bonuses were commonly used, including all the public sector organisations, although in a minority were restricted to senior managers or sales representatives. These were intended to engage staff with organisational success, act as a motivator for achieving organisational objectives or supporting fixed values, e.g. reducing theft or waste through staff vigilance or encouraging productivity. Group bonuses were most visibly seen at MINI amongst production teams; in addition annual bonuses for all staff were noted in ASDA and JLP. Siemens used a combination of individual and team based performance bonuses, biased towards the latter with increasing seniority (due to a greater capability to improve team performance). Although, shop floor staff advised that bonuses, particularly organisational, had a motivational effects and inspired commitment, it was also emphasised that this represented only one factor with culture and values, training and other staff benefits and flexibility at work being of equal importance (JLP-Waitrose). Similarly, recognition rewards were frequently used, particularly in the retail organisations. These were of low monetary value but awarded directly and immediately in response to an action by a member of staff (commonly non-management) acting above and beyond their expected role and on their own initiative. These included gift vouchers, hosted events and organisational awards ceremonies and were publicised internally.

Developing (“building individuals”, (IBM)) and retaining talented staff was also considered a key aim of organisational practice, with staff turnover (and absenteeism) included as a key performance indicator for managers. One public sector organisation used talent profiling early in each employee’s career to identify key skills of potential value to their organisation, work and career (OS). Generic skills-based recruitment was used for lower grade staff in the retail organisations ASDA and JLP-John Lewis, with key skills of team working and communication considered of greater priority than key competencies expected for the role. This possibly reflected that organisations had well established training systems able to train competencies as long as key generic skills were present.

### Values and communication in successful organisations

The use of values in managing staff was common across the organisations but were particularly visible in the commercial and voluntary sectors albeit with different interpretations of their role from guiding principles or defining characteristics of the organisation to directing working practices. Values included statements of the organisations responsibility to their staff and staff responsibility to the organisation. In such cases, staff failing to conform to the values of the organisation was considered a serious and potentially disciplinary issue. Values were visible in varying forms including as domains in staff appraisal, walls of meeting rooms and notice boards and publicly accessible internet sites (see Siemens, [61] IBM, [62] John Lewis Partnership, [63] ASDA [49]).

Values in some organisations described the historical aims and culture of the organisation, in others they were contemporary having been reviewed and updated through a process of staff engagement and consultation. For instance, IBM had undertaken a re-evaluation of its values in 2003 through a virtual discussion with all its staff known as a “Values-Jam” [62]. Similarly the Prince’s Trust undertook an extensive review of its values with consultation with its workforce within planned workshops, leading to culture change and new systems of performance management during its multi-staged and successful turnaround. Although the executive had directed the chosen values, local managers were able to revise their content with respect to local issues and needs. Subsequently its mission statement (“what are we about?....we’re all about young people”) formed the core of its strategy.

Established, structured systems of multidirectional communication between staff and local and corporate management were similarly noted throughout all organisations but were again particularly prominent in the commercial sector (ASDA, JLP-Waitrose). All used a range of methods including electronic staff surveys, “listening group” meetings between staff and managers and staff councils that advised at a senior level (and with constitutional authority in the John Lewis Partnership). This allowed managers to have “visibility” of problems and good practice from a staff perspective as well as engaging with them in problem solving and communicating corporate objectives. For example, at the MINI Plant the assembly line staff regularly reviewed standard operating procedure based processes on discussion and consensus agreement. Furthermore, dedicated process boards were used for discussing problems and solutions. Organisation wide virtual discussion or “Jams” were used in IBM for similarly improving processes and spreading innovation amongst staff that could be geographically distant and working different hours.

Annual staff surveys were used throughout the commercial sector organisations where they were considered an important source of information, providing anonymous staff attitudes of the organisation and its management, including appraising line manager’s capability. This could involve short electronic surveys focusing on 4 or more key organisational issues (as at ASDA) and undertaken by high numbers of staff (e.g. over 90% in one organisation).

The benefits of communication and engagement with staff was most clearly seen in the Prince’s Trust’s change programme in 2004, “One Trust, One Team” that was reviewed through organised staff workshops around the UK, and lead to a number of “quick wins” in improving performance, and aiding the development of strategic priorities set at the regional level. The Prince’s Trust described that “giving staff voice” contributed to a marked increase in staff satisfaction on the annual survey to above 90% and a marked reduction in staff turnover. At a national level, ONS, a public sector organisation undertook a strategy event annually including its executive with 40 senior managers to review the challenges facing the organisation for the year ahead and the most effective planned response. This allowed the benefits of utilising the experience and knowledge of staff just below board level and arguably in greater proximity to their customers, and encouraging both buy-in and dissemination of organisational objectives throughout the organisation.

## Discussion

This study aimed to evaluate and develop a model for HRM of clinicians in speciality practice in secondary healthcare derived from themes representative of best practice across commercial, public and charity sector environments. Comparisons to secondary healthcare were facilitated by the inclusion of organisations that were customer facing, knowledge worker predominant (in common with hospital clinicians), and public sector and/or with a history of organisational development to address changing economic circumstances. The consistency of themes across diverse operating environments supports the “best practice” strategic HRM theoretical framework and although differences were noted between organisations it is unclear whether these were a consequence of their different needs that would support a contingency approach.

From the six themes identified, four directly related to performance management and two, values and communication, to aligning HRM with organisational strategy (e.g. strategic HRM). There were three key approaches to managing performance. Firstly, employee performance *measurement* in the study organisations was undertaken at the team level or above using limited numbers of pre-determined, key performance indicators and including soft intangible measures, for example, customer feedback and the opinions of “mystery shoppers”. Furthermore, managers advised that they used performance data as they felt was most relevant to their roles and needs, without tight coupling to decision making, and with flexibility of interpretation based on their knowledge of other local factors. Secondly, employee performance *improvement* was undertaken at the individual level through formative and line manager led IPR supported by HR departments and aligned to organisational needs and objectives, and with underperformance and recognition and reward strategies. Thirdly, managing the performance, behaviour and development of staff was integrated into organisational strategy and aligned to organisational objectives through the use of universally applied values, supported by investment of time in ensuring communication between staff at the customer interface and the executive. These approaches reflected many of the key aspects of high performance work systems [64] and strategic HRM.

These findings were compared to NHS management practice, where conversely evaluation of clinician performance was proposed at the individual level through “hard systems” of measuring and publically releasing tangible data [6,21,36,65]. Use of performance measures within NHS secondary care at the departmental or organisational levels [33] involved both quantitative and qualitative differences to that noted in the study organisations and included complex and multifaceted targets, e.g. patient waiting times, that are less amenable to interpretation and identifying causality, composite or formulaic analyses, and punitive response and open publication [66] (for public “naming and shaming”). It is argued that this approach associated with a “command and control” centralised style of analysing and addressing performance levels in the NHS [67] has led to a “measurement” culture, contrasting to the stated need for a “performance” culture [59] and resulting in dysfunctional responses from staff, [32,68] gaming of data, [34] and detrimental effects on the quality of patient care [33-35]. This is in addition to the consequences of using performance data that is inaccurate, misinterpreted and misreported [69]. Finally, in place of clear values and lines of communication, clinicians in secondary care organisations are subject to multiple, overlapping and competing systems including their own organisation, local external organisations including commissioning bodies, supra-regional bodies including NHS England and its constituent organisations in workforce planning, education and public health, the department of health, professional bodies, regulatory financial and quality assurance bodies and public inquiries.

In terms of evaluating the performance of individual employees for performance improvement, there were similarities of IPR in the study organisations to clinical appraisal and revalidation in terms of the use of both tangible and intangible outcomes and objective setting [70,71]. However they differed in that IPR was considered as a formative, organisationally lead process undertaken by an individual line manager supported by HR and, in the event that underperformance was identified, leading to an immediate, intensive but supported objective based system of review. The importance accorded to IPR was further emphasised to staff by the dependence of salary increases and bonuses on positive outcomes and the potential for dismissal if underperformance was not addressed. By comparison, clinical appraisal and revalidation of doctors in the NHS is a summative process with formative elements, that is professionally supported but peer lead, to assure the quality of an individual’s practice, and potentially with referral for withdrawal of professional license where this fails [72]. The process of ensuring that a clinician’s performance (including productivity) is in accordance with contractual obligations (e.g. achieving minimum agreed performance levels) is assigned to job planning that may be neither formative nor summative and although organisationally supported is without formal oversight in the NHS other than limited recommendations of professional and governmental bodies [18]. Furthermore, levels of performance identified within job planning and appraisal, and contrasting with noted IPR practice, are not associated with salary increases or other forms of incentivisation [19].

The findings of this study have implications for management of clinicians in NHS secondary care that are perceived as ineffective and leading to disengagement [73-75] and resistance to effective performance measurement, evaluation and improvement [76,77]. This includes that, firstly, there may be a need to review how medical managers are trained and supported and particularly in how they address performance related HRM of their colleagues. Secondly, that there should be an emphasis of performance measurement in the NHS conforming to the proposed requirements for a sustained performance culture and include developing a sense of community and common purpose, honesty and truth about practice, ownership and accountability by staff at all levels as groups and individuals and clarity of what a performance culture entails [78]. This should be considered an inclusive component of the approach to HRM of clinicians. Thirdly, that healthcare performance measurement alone is insufficient to promote sustained and supported productivity improvements, but requires an inclusive human resource management strategy that “nurtures and encourages the pursuit of efficiency” [79] from the clinician treating patients to the executives setting organisational strategy.

Introducing more effective models of employee management using a commercial sector perspective has been a repeated theme in the NHS since the NHS Management Inquiry, 1982 [80]. However, a potential criticism of this study includes conflicting opinion on the value of extrapolating HRM systems from non-healthcare to healthcare organisations particularly where there are recognised differences in the nature of executive management, staff employment and representation, attitude to political changes and the role of profit making [42,81]. Furthermore, it is unknown whether clinicians, as knowledge workers, would respond to similar HRM interventions as noted across the studied organisations. Finally, the heterogeneity of medical subcultures across different organisations suggests that a single HRM model is unlikely to be effective throughout the NHS, indicating the need for local development of this or any HRM framework.

## Conclusion

In conclusion, it may be argued that there has never been a greater need to introduce effective strategies for HRM into the NHS for individual clinicians to transform quality and productivity. These have been introduced in organisations from diverse operating environments that have needed to address the same economic challenges present in the NHS and with an employee base including knowledge workers and in common with secondary healthcare organisations. The results of this study provide a model of co-dependent strategies that may be considered to address this need.

## Abbreviations

NHS: National health service
HRM: Human resource management
PHT: Portsmouth hospitals trust
JLP: John Lewis partnership
MINI: The MINI plant, Oxford
ONS: Office for national statistics
NATS: National air traffic services
OS: Ordnance survey
